# Availability and Key Characteristics of National Early Warning Systems for Emerging Profiles of Antimicrobial Resistance in High-Income Countries: Systematic Review

**DOI:** 10.2196/57457

**Published:** 2025-01-15

**Authors:** Jessica Iera, Claudia Isonne, Chiara Seghieri, Lara Tavoschi, Mariateresa Ceparano, Antonio Sciurti, Alessia D'Alisera, Monica Sane Schepisi, Giuseppe Migliara, Carolina Marzuillo, Paolo Villari, Fortunato D'Ancona, Valentina Baccolini

**Affiliations:** 1 Department of Public Health and Infectious Diseases, Sapienza University of Rome, Rome, Italy; 2 Department EMbeDS, Sant'Anna School of Advanced Studies, Management and Health Laboratory, Institute of Management, Pisa, Italy; 3 Department of Infectious Diseases, Istituto Superiore di Sanità, Rome, Italy; 4 Department of Translational Research and New Technologies in Medicine and Surgery, University of Pisa, Pisa, Italy; 5 Italian Ministry of Health, General Directorate for Health Prevention, Rome, Italy; 6 Department of Life Sciences, Health and Health Professions, Link Campus University, Rome, Italy

**Keywords:** early warning system, surveillance, emerging AMR, high-income countries, antimicrobial resistance

## Abstract

**Background:**

The World Health Organization (WHO) recently advocated an urgent need for implementing national surveillance systems for the timely detection and reporting of emerging antimicrobial resistance (AMR). However, public information on the existing national early warning systems (EWSs) is often incomplete, and a comprehensive overview on this topic is currently lacking.

**Objective:**

This review aimed to map the availability of EWSs for emerging AMR in high-income countries and describe their main characteristics.

**Methods:**

A systematic review was performed on bibliographic databases, and a targeted search was conducted on national websites. Any article, report, or web page describing national EWSs in high-income countries was eligible for inclusion. EWSs were identified considering the emerging AMR-reporting WHO framework.

**Results:**

We identified 7 national EWSs from 72 high-income countries: 2 in the East Asia and Pacific Region (Australia and Japan), 3 in Europe and Central Asia (France, Sweden, and the United Kingdom), and 2 in North America (the United States and Canada). The systems were established quite recently; in most cases, they covered both community and hospital settings, but their main characteristics varied widely across countries in terms of the organization and microorganisms under surveillance, with also different definitions of emerging AMR and alert functioning. A formal system assessment was available only in Australia.

**Conclusions:**

A broader implementation and investment of national surveillance systems for the early detection of emerging AMR are still needed to establish EWSs in countries and regions lacking such capabilities. More standardized data collection and reporting are also advisable to improve cooperation on a global scale. Further research is required to provide an in-depth analysis of EWSs, as this study is limited to publicly available data in high-income countries.

## Introduction

Antimicrobial resistance (AMR) is a global public health emergency [1,2] that increases the number of deaths, length of hospital stays, and health care costs expenditure [3-8]. Worldwide, a total of 4.95 million deaths per year are associated with bacterial AMR, 1.27 million of which are directly attributable to it [9]. Gram-negative bacteria resistant to last-resort antibiotics such as carbapenem-resistant *Acinetobacter baumannii* and carbapenem-resistant and third-generation cephalosporin-resistant *Enterobacterales* have been listed as of critical priority for public health measures, due to their significant global burden and the ability to transfer resistance genes [10]. As the threat of AMR continues to grow, more efforts are needed to strengthen infection prevention and control strategies as well as to enhance surveillance systems [11].

AMR surveillance plays a pivotal role in strengthening health system resilience and preparedness, thereby supporting effective antibiotic stewardship and leading to optimized patient health outcomes [12]. Enhanced investments are considered essential to improve the comparability, quantity, and quality of AMR data [13]. Current trends, such as the rising prevalence of carbapenem-resistant *Acinetobacter spp*. isolates, underline the need to intensify efforts for the early detection of drug resistance [13]. Several international initiatives aimed at improving epidemiological and microbiological discussion for coordinated actions are ongoing [14,15]. Within this context, the World Health Organization (WHO) launched the Global Antimicrobial Resistance and Use Surveillance System (GLASS) in 2015 [16,17], included the development of national surveillance systems capable of timely detecting and reporting emerging resistance among the goals outlined in the Global Plan of Action on AMR published in 2016 [18], and more recently, developed a focused surveillance with a specific reporting component for novel (emerging) AMR (ie, GLASS-EAR), in order to support early data sharing and coordinated actions among member states [19].

However, while well-established routine AMR surveillance systems are available in high-income countries (HIC), implementing AMR surveillance has been particularly challenging in low- and middle-income countries, where the number of surveillance sites contributing to national surveillance is often not representative [20,21], and a limited number of referral hospitals report AMR data to GLASS [22]. Therefore, studies have mainly focused on countries where the laboratory capabilities required to strengthen national surveillance systems are more likely to be in place (ie, HIC) and publicly available information on the implementation of tools for the early detection and reporting of emerging AMR profiles is often fragmented [23]. Even when findings are available, understanding these systems is challenging due to different approaches used for data collection, reporting, and interpretation of definitions [24,25]. Given the lack of a clear and comprehensive overview on the topic, the aim of our study was to map existing national early warning systems (EWSs) for emerging AMR in HIC as well as describe and compare their main characteristics. Findings from our study could provide relevant information to stakeholders engaged in AMR surveillance, informing the development and/or optimization of EWSs in several countries, including low- and middle-income countries.

## Methods

### Ethical Considerations

This study was performed according to the Cochrane Handbook for Systematic Reviews and the PRISMA (Preferred Reporting Items for Systematic Reviews and Meta-Analyses) statement (Checklist 1) [26,27]. The protocol was registered on the Open Science Framework via the Center for Open Science (210 Ridge McIntire Road, Suite 500, Charlottesville, VA 22903‐5083; identifier: pr6a8). As the study did not involve the collection of primary data, it did not require informed consent or the submission for institutional review board approval.

### Search Strategy and Study Selection

Three bibliographic databases (ie, PubMed, Web of Science, and Scopus) were searched. Search strings were adapted to fit the search criteria of each database (Table S1 in Multimedia Appendix 1). Taking into account the topic under investigation and the expected challenges in retrieving information, we intentionally developed broad search strings, prioritizing sensitivity over specificity. The search was conducted among records published from database inception to July 3, 2024, without restrictions such as language or date. After the removal of duplicate articles, language restriction was applied in the context of the title and abstract of all the retrieved records during screening, according to the eligibility criteria described below. The full texts of potentially relevant articles were examined by three researchers, who resolved disagreements through discussion and recorded reasons for exclusion. In addition, relevant national websites (ie, the Ministries of Health and/or National Health Institutes websites) were also explored, focusing on thematic areas involving AMR surveillance (Table S2 in Multimedia Appendix 1).

### Eligibility Criteria

Considering the Emerging Antimicrobial Resistance Reporting framework within the GLASS developed by the WHO [19], EWSs were defined, for the purposes of this study, as any system able to timely detect, provide verification, and report emerging AMR events. This broad definition was adopted as we expected to encounter systems with diverse characteristics, including variations in design, data flow requirements, implementation, and functionality. Emerging AMR was defined as unusual AMR findings in bacteria and fungi causing infections in humans with the potential impact on public health, such as new types of phenotypic resistance (not previously reported or very rare) and new genetic determinants of AMR that may have high potential for spread. As a specific resistant phenotype/genotype may be emerging in one area but may already be endemic in another one, a list of AMR events was not developed by considering both newly detected AMR and known resistance profiles detected for the first time in new geographical areas.

We included articles, web pages, and/or technical reports with the following characteristics: (1) reported in English, French, Spanish, or Italian, aiming at broadening our investigation by taking into account the language capabilities of the co-authors; (2) describing national EWSs for emerging AMR currently implemented or under development; and (3) referring to HIC, as identified by the World Bank classification of countries by income [28]. We excluded articles, web pages, and/or technical reports that (1) described only standard national AMR surveillance systems, for example routine surveillance systems that retrospectively analyzed data on an annual basis and/or that lacked active alert components; (2) focused on EWSs limited to subnational levels; or (3) reported data in languages other than English, French, Spanish, or Italian.

### Data Collection and Synthesis

For each record, two reviewers independently retrieved the following information using a standardized data abstraction form: identification of the report/study, if applicable (title, first author, year of publication, and DOI); country; key characteristics of the EWS (eg, coordinating institution, year of establishment, sector involved—human/animal/food/environment, setting where specimens are collected, specimen types, and availability of genomic data); key characteristics of the microorganisms under surveillance (eg, resistance profiles); alert timing (eg, real-time, daily, or weekly); and conduction of performance evaluation of the EWS. Countries were grouped by region, according to the World Bank classification. Data were descriptively synthesized through tables and graphical representations using R software (version 4.3.1; R Foundation for Statistical Computing).

## Results

### Records Identification and Screening

**Figure 1.**
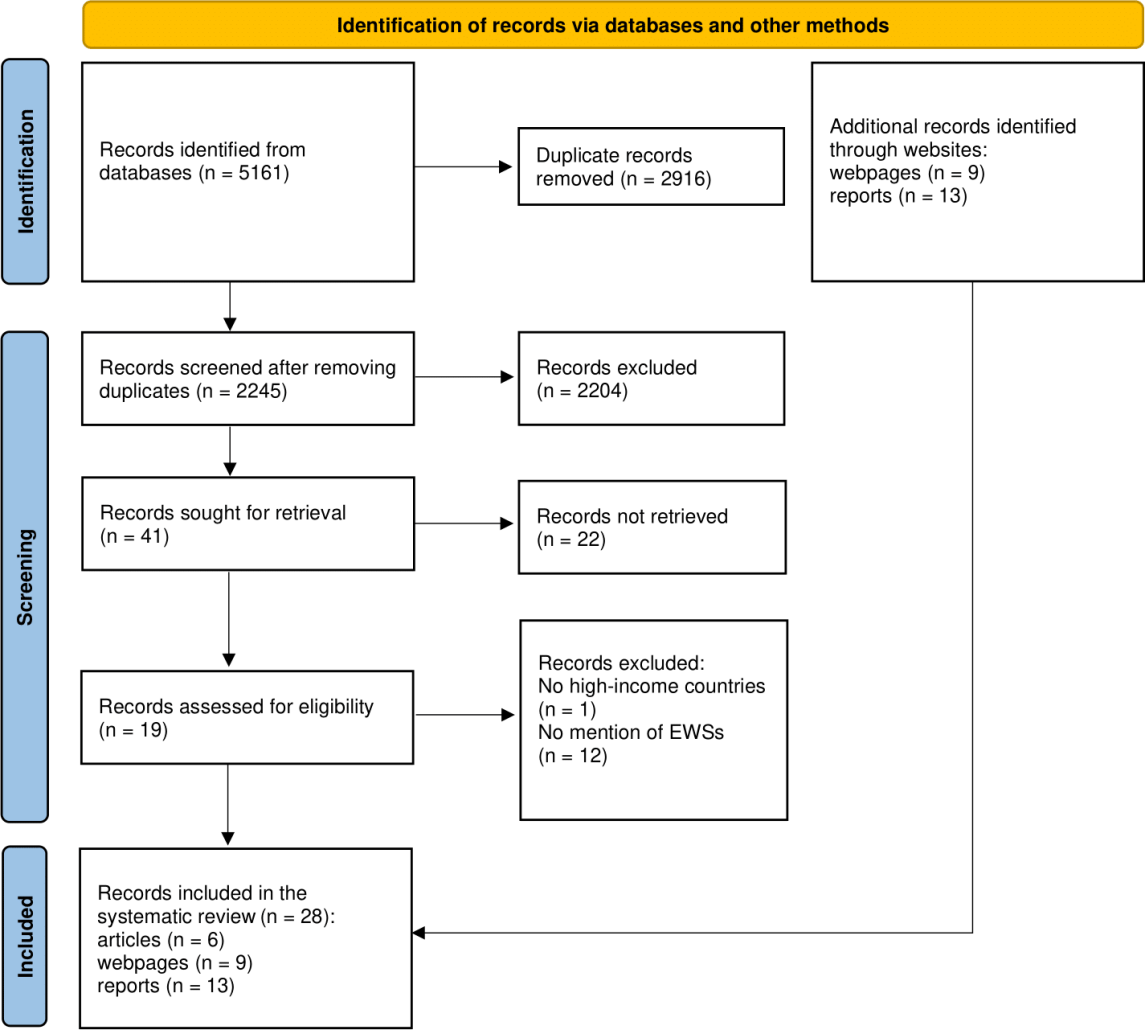
PRISMA (Preferred Reporting Items for Systematic Reviews and Meta-Analyses) flow diagram of the review process. EWSs: early warning systems.

Overall, 5161 records were identified via database searching (Figure 1). After duplicate removal and screening by the title and abstract, 41 articles were eligible for full-text analysis. Of these, 35 were excluded with reasons, providing a total of 6 articles ultimately included in the systematic review [24,29-33]. A targeted search on relevant national websites allowed for the identification of thematic areas involving AMR from 72 out of 86 HIC (15 countries were excluded due to language restrictions or the inability to retrieve websites), with 22 records finally included in the systematic review (ie, 9 web pages [34-42] and 13 reports [25,43-54]).

### Availability of National EWSs for Emerging AMR in HIC

#### Overview

**Figure 2.**
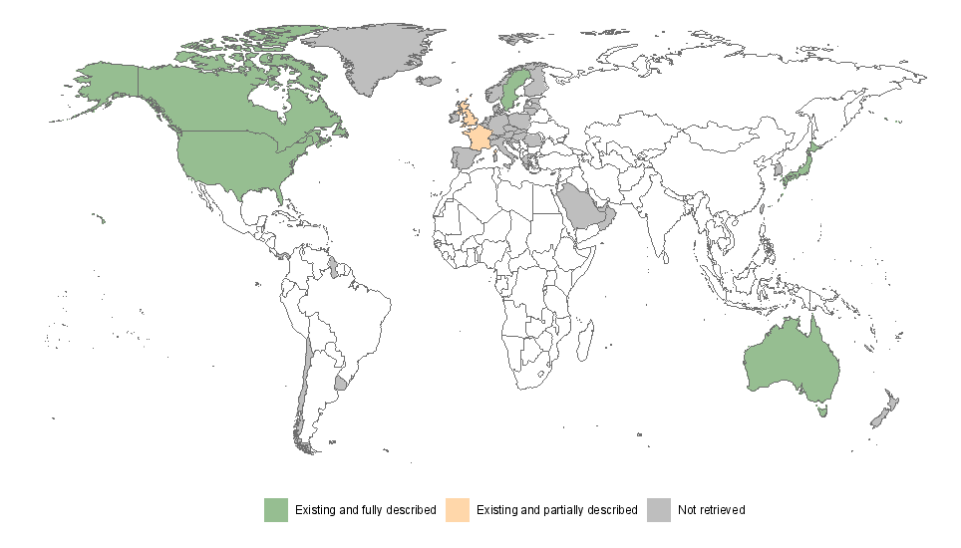
Availability of early warning systems for emerging antimicrobial resistance at the national level in high-income countries.

Nine web pages and 19 publications described 7 national EWSs in 72 (9.7%) HIC (Figure 2). Of these, 2 were in the East Asia and Pacific Region (ie, Australia [29,34,44,45,53,54] and Japan [24,30,31,33]), 3 were in Europe and Central Asia (ie, France [36-38,48,49], Sweden [24,39,51,52], and the United Kingdom [32,40,46]), and 2 were in North America (ie, United States [25,41,42,50] and Canada [35,43,47]). A partial description of the systems was available in 2 cases (ie, the United Kingdom and France).

#### East Asia and Pacific Region

##### Australia: National Alert System for Critical Antimicrobial Resistances

The National Alert System for Critical Antimicrobial Resistances (CAR-Alert) was established by the Australian Commission on Safety and Quality in Health Care in March 2016 as part of the Antimicrobial Use and Resistance in Australia (ie, AURA) Surveillance System and reviewed in 2018 and 2022 (Table 1) [54]. By collecting data on nationally agreed priority microorganisms with critical resistance to last-line antimicrobial agents, it is a coordinated national system that allows both the collection and the communication of information on confirmed critical antimicrobial resistance (CAR) and potential outbreaks, as close as possible to the time of confirmation [29,34]. At the national level, 28 confirming laboratories provided data to the system in 2021 and 2022, with at least 1 confirming laboratory in each state and territory [53]. In 2023, 26 confirming laboratories participated in the CAR-Alert [54].

**Table 1.** National EWSs^a^ for emerging AMR^b^ identified in high-income countries.

Country	EWS	Institution	Year of establishment	Brief description
East Asia and Pacific Region				
Australia	CAR-AlertNational Alert Systemfor Critical AntimicrobialResistances	Australian Commission on Safety and Quality in Health Care	2016Revised in 2018 and 2022	CAR-Alert allows for the timely surveillance of critical nationally agreed antimicrobial resistances, integrating the AURA^c^ surveillance system.The system involves laboratories conducting confirmatory susceptibility testing on human isolates.
Japan	JANIS-CLJapan nosocomial infection surveillance-Clinical Laboratory division	National Institute of Infectious Diseases	2000Revised in 2007	The system involves several laboratories across the country. Alert emails are automatically sent to contact personnel of facilities that report unusual combinations of bacterial species and antimicrobial susceptibility, defined as “unusual AMR.”
North America				
Canada	AMRnetCanadian AntimicrobialResistance Network	Public Health Agency of Canada	NA^d^	Laboratory-based surveillance system capturing information on AMR susceptibility testing from laboratory information systems in both public and private clinical and veterinary laboratories, including reference laboratories. The system aims at collecting and disseminating real time data to allow stakeholders response to emerging AMR issues.
United States	AR Lab NetworkAntimicrobial Resistance Laboratory Networks	Center for Disease Control and Prevention	2016	Provides nationwide lab capacity to rapidly detect antimicrobial resistance. Laboratories cooperate to rapidly identify AMR profiles causing hard-to-treat or potentially untreatable infections. Protocols to immediately notify health department, health care provider, and infection control staff of unusual resistance are provided.
Europe and Central Asia				
France	e-SINSignalement Externe des Infections Nosocomiales	Ministry of Health	2011Revised in 2017	Application that allows the monitoring of infectious events in health care facilities, providing data on isolated cases/outbreaks of bacteria with particular AMR profiles. A specific reporting form for emerging highly resistant bacteria is available since 2017. The National Reference Center for Antibiotic Resistance is integrated into this alert system, in particular for events involving emerging mechanisms of resistance. Reports may result in national or international health alerts.
Sweden	Svebar	Public Health Agency of Sweden	2015	Svebar consists of an IT system for early alerts and continuous resistance monitoring. All culture findings from the country’s laboratories are automatically transferred on a daily basis, allowing an early warning on findings of serious antibiotics resistance.
United Kingdom	Antimicrobial resistance alerts (ARAs)	Public Health EnglandAntimicrobial resistance and healthcare associated infections reference unit (AMRHAI)	NA	AMRHAI detects new and emerging AMR via interpretive analysis of MIC^e^ profiles/molecular investigation. Unusual isolates are sent for testing from diagnostic laboratories to AMRHAI, where an appropriate reference unit confirms relevant isolates resistance. If deemed of public health impact, ARAs inform microbiologists of emerging AMR pathogen strains that could spread in the United Kingdom health service.

a EWS: early warning system.

b AMR: antimicrobial resistance.

c AURA: Antimicrobial Use and Resistance in Australia.

d NA: not available.

e MIC: minimum inhibitory concentration.

In order to identify and confirm potential CAR, pathology laboratories perform a routine three-step process based on (1) isolate collection from the patient and routine testing; (2) confirmation by a laboratory with the capacity to identify CAR, if a critical AMR profile is suspected by the originating laboratory; and (3) data submission to the CAR-Alert web portal by the confirming laboratory and feedback to the originating laboratory, allowing the implementation of proper infection prevention and control by the health service caring for the patient [44,45].

##### Japan: Japan Nosocomial Infections Surveillance - Clinical Laboratory Division

The Ministry of Health, Labour, and Welfare of Japan provided two national surveillance systems to monitor AMR in bacteria, including the Japan Nosocomial Infections Surveillance (ie, JANIS) (Table 1) [31]. JANIS was launched in 2000 as a voluntary surveillance system focusing on infections in health care settings and includes several divisions [30,33]. Among them, the JANIS clinical laboratory division (JANIS-CL) specifically focuses on AMR bacteria, and it collects comprehensive specimen-based data from participating hospitals on a monthly basis through a member-restricted website. Submitted files from participating hospitals are automatically processed to check data structure, interpret antimicrobial susceptibility, remove duplicates, and calculate the prevalence of AMR [30]. The system has the capacity to detect unusual combinations of bacterial species and antimicrobial susceptibility, defined as unusual AMR, and generates alert emails that are automatically sent to the contact personnel of the facilities [31].

### North America

#### Canada: Antimicrobial Resistance Network

The Antimicrobial Resistance Network (AMRNet) is a collaboration between the Public Health Agency of Canada, provincial and territorial public health organizations, and clinical and veterinary laboratories across the country (Table 1). The system has been designed to detect the emergence and spread of AMR and to timely disseminate data to stakeholders, in order to address emerging AMR issues in Canada [35,47].

Originally started as a series of pilot projects, the AMRNet transitioned to collecting routine data (antimicrobial susceptibility testing results from bacterial and fungal pathogens, together with a defined set of patient or animal characteristics) from a select group of provinces in 2022. Currently, data collection involves approximately 1.5 million bacterial and fungal isolates per year from Ontario, Saskatchewan, and Prince Edward Island [43].

#### United States: The Antimicrobial Resistance Laboratory Network

The Antimicrobial Resistance Laboratory Network (AR Lab Network) was established and is coordinated by the Centers for Disease Control and Prevention (CDC) to rapidly detect existing and emerging resistance nationwide (Table 1). The network includes 7 regional laboratories, the National Tuberculosis Molecular Surveillance Center, and laboratories across 50 states, 5 cities, and Puerto Rico [25,50]. Laboratories cooperate, from the local to the national level, to provide the rapid identification and response to antimicrobial-resistant genes and microorganisms that cause hard-to-treat or potentially untreatable infections. When unusual resistance is detected, the Healthcare and Clinical Laboratories notify the Health Department. Public health laboratories then confirm the bacterial species identity, perform additional testing to characterize the isolates received, provide notification of unusual resistance to CDC and, when appropriate, send the isolates to regional laboratories for additional testing [42]. Every regional laboratory performs core testing, including the detection of new and emerging threats. If needed, regional laboratories send unusual isolates to the CDC to confirm testing and conduct additional tests. When threats are reported, the CDC provides infection prevention and control recommendations and supports outbreak responses [41].

### Europe and Central Asia

#### France: E-SIN (Signalement Externe Des Infections Nosocomiales - External Reporting on Healthcare-Associated Infections)

Since 2011, the reporting of health care–associated infections, initially introduced in 2001, has been based on a digital tool called e-SIN (Signalement Externe Des Infections Nosocomiales - External Reporting on Healthcare-Associated Infections; Table 1) [48]. The e-SIN application monitors infectious cases reported by health care facilities and allows for the identification of national or even international health alerts, thereby providing data on isolated cases or outbreaks of bacteria with exceptional AMR profiles such as emerging highly resistant bacteria (defined as microorganisms with susceptibility to only one or two classes of antibiotics) [37,38,49]. The reporting process, moreover, enables a simultaneous reporting to both the Regional Coordination Centers for Healthcare-Associated Infections and the Regional Health Agency and includes an internal alert system that allows the notification of the members of the Epidemiology and Hospital Hygiene Team [36].

#### Sweden: Svebar

The Public Health Agency of Sweden is responsible for national AMR monitoring and analysis (Table 1) [39]. The IT system Svebar was developed in 2015 to expand and strengthen existing surveillance systems both at the national and local levels [24,52]. This system relies on the voluntary participation of clinical microbiology laboratories. All data on clinical isolates from humans are transferred to Svebar by connected laboratories on a daily basis. Every night, the participating laboratories automatically send to the system a file, saved in a short-term storage, with the culture findings from the past 14 days. Data are reported according to standard definitions and saved in a short-term storage for 13 days before being transferred to a long-term storage, which retains the data from the previous night’s report, thereby receiving more processed data [51]. The system allows the generation of early alerts and the continuous monitoring of serious antibiotic resistance or other undesired changes in resistance conditions, through pre-set alert functions [51]. Currently, 22 laboratories provide data to Svebar [52].

#### United Kingdom: Antimicrobial Resistance Alerts

The UK antimicrobial resistance alerts (ARAs) provide microbiologists with information of emerging antimicrobial-resistant pathogen strains that could spread in the UK health service (Table 1) [32]. When a suspicious resistant strain is identified, isolates are sent to Public Health England’s AMRHAI reference unit for further testing. The AMRHAI reference unit includes the national reference laboratory for AMR investigation and detection of new and emerging resistances: the unit investigate isolates found to have unusual resistances by diagnostic laboratories, aiming to identify emerging resistance of public health importance, underlying resistance mechanisms and clonal spread of resistant strains [46].

Proposals for ARAs can be submitted by any interested party and should be directed to the co-chairs of a standing committee, which review the proposals and determine its public health implications, in order to assess the necessity of issuing a national alert [40].

### Characteristics of National EWSs for Emerging AMR in HIC

#### Health Sectors Involved, Specimen Type, and Setting

The human sector was involved in all the identified EWSs, and, in the case of Canada, the system also included the animal sector [47]. All the systems reported data from clinical isolates, while 2 also included screening samples (ie, Australia and the United States; Table 2) [45,50].

**Table 2.** Main characteristics of national early warning systems for emerging AMR^a^ in high-income countries.

Country	Sector	Setting	Specimen type	AMR profiles of microorganisms	Genomic data	Alert timing	Performance evaluation
East Asia and Pacific Region							
Australia (CAR-Alert)	Human	Hospital-basedCommunity-based	Clinical isolatescreening	Carbapenemase-producing *Acinetobacter baumannii* complex *Candida auris* Carbapenemase-producing *Enterobacterales* and/or ribosomal methyltransferase-producing *Enterobacterales*, with transmissible colistin-resistance Linezolid-resistant *Enterococcus* species Multidrug-resistant *Mycobacterium tuberculosis* – resistant to at least rifampicin and isoniazid Ceftriaxone- and/or azithromycin-nonsusceptible *Neisseria gonorrhoeae*; gentamicin-resistant *Neisseria gonorrhoeae* Ciprofloxacin-nonsusceptible *Neisseria meningitidis* Carbapenemase-producing *Pseudomonas aeruginosa* Ceftriaxone-nonsusceptible *Salmonella* species Multidrug-resistant *Shigella* species Vancomycin- or linezolid-nonsusceptible *Staphylococcus aureus* complex (*S argenteus* and *S schweitzeri*) Penicillin-reduced susceptibility *Streptococcus pyogenes*	Yes	Weekly summary alert emails	Yes
Japan (Janis-CL)	Human	Hospital-basedCommunity-based	Clinical isolates	AMR never officially reported (eg, vancomycin-resistant *Staphylococcus aureus*) AMR reported but rare (eg, multidrug-resistant *Acinetobacter spp*. and vancomycin-resistant enterococci)	Yes	Alert emails within 1‐2 h	NA^b^
North America							
Canada (AMRnet)	HumanAnimal	Hospital-basedCommunity-based	Clinical isolates	All bacterial and fungal pathogens tested for AMR, including priority pathogens^c^, as classified by the World Health Organization	NA	NA	NA
United States (AR Lab Network)	Human	Hospital-basedCommunity-based	Clinical isolatescreening	Carbapenemase-producing organism (CPO), including carbapenem-resistant *Enterobacterales* (CRE) *Candida* species, to identify emerging resistance Emerging and concerning threats, such as mcr-1 and carbapenem-resistant *Acinetobacter baumannii*, and changes in known threats, including MRSA Pandrug-resistant (PDR) bacteria to new antibiotics	Yes	NA	NA
Europe and Central Asia							
France (e-SIN)	Human	Hospital-based	Clinical isolates	Carbapenemase-producing *Enterobacterales* Glycopeptide-resistant *Enterococcus faecium*	NA	NA	NA
Sweden (Svebar)	Human	Hospital-basedCommunity-based	Clinical isolates	Bacterial species resistant to one or more antibiotics (ie, *E coli* resistant to carbapenems) A trend (ie, resistance to ampicillin in more than 30 percent of cases of *E coli*).	Yes	Daily	NA
United Kingdom (ARAs)	Human	Hospital-based	Clinical isolates	The AMRHAI^d^ advises the referral of isolates with exceptional resistance phenotypes. A list of unusual combinations of resistance and organisms is available for diagnostic laboratories^e^, including the following microorganisms: *Acinetobacter spp*. Coagulase-negative *staphylococci* *Enterobacterales* *Enterococci* *Haemophilus influenzae* *Moraxella catarrhalis* Organisms or antibiotics for which there are no EUCAST^f^ clinical breakpoints (invasive sites) *Pseudomonas aeruginosa* *Staphylococcus aureus* *Streptococci* (groups A, B, C, and G, β-haemolytic) *Streptococcus pneumoniae*	Yes	NA	NA

a AMR: antimicrobial resistance.

b NA: not available.

c *Acinetobacter* spp; *Escherichia coli*; *Klebsiella pneumoniae*; *Neisseria gonorrhoeae*; *Salmonella* spp; *Shigella* spp; *Staphylococcus aureus*; *Streptococcus pneumoniae*.

d AMRHAI: antimicrobial resistance and healthcare-associated infections reference unit.

e United Kingdom Health Security Agency Reference Laboratories Colindale. Bacteriology Reference Department user manual. Appendix 1 (Antimicrobial resistance and mechanisms service). Version 15, October 10, 2022.

f EUCAST: European Committee on Antimicrobial Susceptibility Testing.

The setting where specimens are collected was hospital based and community based in all the identified systems except for e-SIN (France) and ARAs (United Kingdom), where the collection was mainly hospital based [36,46]. More specifically, CARAlert (Australia) was the most comprehensive alert system, including public and private hospitals, general practices, aged care homes, community health services, and hospital nonadmitted care services [54]; JANIS-CL (Japan) included outpatients and inpatients data from large (usually tertiary care) and small hospitals (usually privately owned hospitals with less than 200 beds, providing both acute and long-term care) [31]; and AMRnet (Canada) included AMR data from clinical laboratories in both public and private facilities [43]. Detailed published information was not available referring to the remaining EWSs.

#### AMR Events Reported and Availability of Genomic Data

Reported AMR events varied widely among the identified EWSs (Table 2). In the majority of instances, emerging AMR was classified in terms of broader categories of unusual events (eg, AMR never previously officially reported, AMR reported but rarely, or a trend). Notably, among the 7 systems examined, 3 systems (ie, Australia, France, and the UK) provided a list of unusual combinations of microorganisms and resistance, including in most cases, carbapenemase-producing *Acinetobacter baumannii* and carbapenemase-producing *Enterobacterales* [38,44,46]. Two countries (ie, Australia and the United States) included emerging fungal pathogens such as *Candida auris* in their systems [25,44].

Genomic data were provided by 5 out of 7 EWSs, while this information was not retrievable for 2 countries, specifically Canada and France [25,33,44,46,52].

#### Alert Functions

Alert timing and functioning were clearly defined in 3 out of 7 EWSs (ie, Australia, Japan, and Sweden), with several differences among these systems. Concerning CAR-Alert (Australia), data are submitted within 7 days of the isolate being confirmed as a CAR (Table 2). The system generates a weekly summary email alert to report information on confirmed CARs to state and territory health authorities, the Australian Government Department of Health, and confirming laboratories, supporting timely responses to CARs by hospitals and state and territory health departments [45].

In the case of JANIS-CL (Japan), a data format was developed as a unified standard for collecting electronic data from microbiology laboratories. Microbiological alerts can be checked on the JANIS member-restricted website within a few hours after data submission. Furthermore, alert emails reporting unusual profiles of AMR are automatically sent within 1 or 2 hours to facilities contact personnel while monthly feedback emails are sent within 48 hours [30].

Regarding Svebar (Sweden), preset alerting algorithms search through the short-term storage and generate an alert in specific conditions, for example when the system discovers a trend. National alerts are sent via e-mail to the contact person at the local laboratory and to administrators at the Public Health Agency of Sweden, and if needed, they can discuss the alert, while local alerts only reach the contact person in the local laboratory. By systematically tracking the progression of resistance, the detection of early-stage changes might justify modifications to the alert system’s settings [51].

#### Performance Evaluation

Based on publicly available information, the formal assessment of national EWSs has been conducted in only 1 country, specifically Australia, where the Department of Health and Aged Care maintains a routine process of evaluating national surveillance systems to ensure they align with their intended purpose and objectives (Table 2). The evaluation of CAR-Alert took place in 2022‐2023, with the aim of assessing the system’s operational efficiency in fulfilling its objectives, evaluating their appropriateness, and identifying potential enhancements to improve the system’s ability to achieve its goals [54].

To assess the system’s overall effectiveness and performance, the evaluation process was based on the “Updated guidelines for evaluating public health surveillance systems” from the CDC [55].

## Discussion

### Principal Findings

Our systematic review allowed the identification of national EWSs for emerging profiles of AMR in a limited number of HIC, approximately 10%. In addition, only half of the 6 World Bank regions that we investigated had functional EWSs in place, revealing a disparity in the availability of these systems at the global level. Given that several regions, including Latin America and the Caribbean, are currently facing alarming developments with national authorities raising concerns about the emergence of carbapenemase-producing *Enterobacterales* not previously described, or about the increasing number of isolates that coexpress two or more of these enzymes [56], the lack of available information on national EWSs in these countries is concerning. Even in the European region, where countries are mostly high-income and have advanced surveillance and health care systems, national EWSs were not retrievable in those areas where AMR rates have been found to be extremely high by the European Center for Disease Prevention and Control (ECDC) [13]. Such an absence of EWSs in high AMR burden countries reveals a critical gap in the region’s preparedness and response to the growing challenge of AMR [54], making it difficult for these nations to not only track the evolution of resistance patterns over time, but also early detect and mitigate the spread of unusual resistant bacteria, implement targeted interventions in high-risk settings, and contribute to shared data on a global scale [19]. In 2021, the ECDC launched an online portal (EpiPulse) for European public health authorities, aiming at enhancing the early detection and assessment of threats due to infectious diseases. However, its effectiveness relies on information from member states concerning microbiological alerts, and without national EWSs in place, the management of threats at the international level becomes challenging [15].

### Comparison With Previous Studies

Notably, while identified EWSs were established quite recently and shared the overarching goal of monitoring and responding to emerging AMR threats, our study showed that their main characteristics varied significantly across countries. This heterogeneity might be attributable to several factors, including the unique health care infrastructure, different public health policies and funding, and the specific challenges and priorities faced by each country [57-59]. The heterogeneity in identified EWSs also reflects the specific AMR trends and epidemiological situation within each state, considering that AMR profiles can vary significantly, being unusual in one country and endemic in another [60]. Nevertheless, some common ground in monitoring, understanding, and responding to AMR emerged in a few aspects, especially if we consider the inclusion of the community setting and the availability of genomic data in most of the identified EWSs. Several reasons can explain this approach: community-based surveillance is crucial because resistant microorganisms can spread within health care and community settings, with early detection allowing the proper prevention and control of outbreaks [61], while genomic data play a pivotal role in understanding AMR trends and developing targeted prevention and control interventions [62]. As for the availability of performance evaluation of EWSs, which is crucial to assess their effectiveness, we found that Australia was the only country known to conduct a system evaluation in accordance with the CDC guidelines, even if related data are not yet available [54]. Considering that regular evaluations can lead to improvements in the system design, data collection, and response mechanisms, and ultimately enhance the ability to respond effectively to emerging AMR profiles [55], more efforts should be made to institutionalize regular assessments as an integral part of any strategy aimed to manage AMR.

Lastly, although the existence of EWSs for emerging AMR was not clearly reported in most cases, this review found that many countries indicated, in their National Action Plans against AMR, the goal of strengthening national surveillance through the implementation of these systems in the next years. This is comforting, as this underlines their commitment in addressing this issue, in line with the WHO recommendations [18,63-65]. Furthermore, as the landscape of infectious disease surveillance is rapidly evolving owing to the advancements in new technologies, there might be a significant potential for progress in bolstering the efforts against emerging AMR [66]. Artificial intelligence, for example, could enhance AMR surveillance systems given its ability to integrate and analyze data from various sources, such as clinical data and microbiology reports, recognize AMR patterns, develop predictive models that signal the early emergence of resistant strains or the spread of resistance in specific regions or health care facilities, or provide real-time insights into AMR trends [67]. The incorporation of machine learning algorithms to predict trends in resistance development based on historical data could also improve AMR surveillance systems capacity in early warning [68]. Therefore, as artificial intelligence technologies continue to improve, they are definitely an option to be considered as a supporting tool in the early detection of emerging AMR in the near future, enabling a more timely and effective response also by facilitating the sharing and analysis of data on a global scale [69].

### Limitations

To the best of our knowledge, this is the first synthesis review of publicly available information on national EWSs for emerging AMR in HIC. The limitations of our study mostly rely on the inclusion criteria we adopted. First, by focusing on human surveillance, our study did not include other relevant sectors in the context of the “One Health” approach [70,71]. Second, by limiting evidence collection to HIC, we did not address challenges faced by countries, such as low- and middle-income countries, where AMR has a disproportionate impact due to higher burden of infections, reduced laboratory capabilities, and limited regulations involving antimicrobial use [72] Third, the restriction to the national level did not account for regional or local-level EWSs, which could be available in some countries [23]. However, the decision to focus on national rather than subnational EWSs relies on the aim of mapping systems capable of centralizing AMR-related data through a comprehensive and coordinated approach, facilitating global data sharing and response strategies, in accordance with the WHO recommendations [18]. Moreover, we are aware that, as this study relies on publicly available information, and not all EWSs might be documented or publicly disclosed [73], our review may not fully represent the extent of EWSs across different countries and across various levels of implementation, such as national and subnational levels. Similarly, the partial availability of information on a few systems limited our ability to fully describe the different strategies adopted in the investigated areas. Nevertheless, our interest was focused on mapping the efforts made by countries at the national level, and we simultaneously searched multiple data sources as an attempt to retrieve all available information. Findings from our study could support countries currently lacking EWSs in the process of strengthening national surveillance systems and contribute to global effort, through improved detection, reporting, and data sharing, in tackling AMR. Additional information is certainly required to provide a complete overview on available EWSs. To this end, our review could be integrated with a country consultation and/or international survey aimed at retrieving additional findings from relevant stakeholders, such as the Ministries of Health and National Health Institutes representatives. Further investigations could also provide an in-depth analysis on the availability of EWSs at subnational (local or regional) levels and in low- and middle-income countries.

### Conclusions

These findings highlight the urgent need for a broader implementation of surveillance systems that allow for the early detection of emerging AMR, with increased investments and collaborative efforts to establish EWSs in countries and regions lacking such capabilities to date. Platforms such as EpiPulse within the European region could enhance the collection and analysis of AMR surveillance data, taking into account that each country should implement tools capable of detecting and managing alert signals. In addition, given the heterogeneity in national health care systems and their different epidemiological contexts, tailored approaches that enable the collection of standardized and comparable AMR data are strongly encouraged to help promote the global preparedness for AMR.

## Supplementary material

10.2196/57457 Multimedia Appendix 1 Search strategies used in the systematic review and high-income countries’ institutional websites explored.

10.2196/57457 Checklist 1 PRISMA (Preferred Reporting Items for Systematic Reviews and Meta-Analyses) checklist.

## Abbreviations

AMR: antimicrobial resistance
AMRNet: Antimicrobial Resistance Network
AR Lab Network: Antimicrobial Resistance Laboratory Network
ARAs: antimicrobial resistance alerts
CAR: critical antimicrobial resistances
CAR-Alert: National Alert System for Critical Antimicrobial Resistances
CDC: Centers for Disease Control and Prevention
ECDC: European Center for Disease Prevention and Control
EWSs: early warning systems
GLASS: Global Antimicrobial Resistance and Use Surveillance System
HIC: high-income countries
JANIS-CL: Japan Nosocomial Infections Surveillance - Clinical Laboratory Division
PRISMA: Preferred Reporting Items for Systematic Reviews and Meta-Analyses
WHO: World Health Organization

## References

[1] Antimicrobial resistance. World Health Organization.

[2] About antimicrobial resistance. Centers for Disease Control and Prevention (CDC).

[3] O’Neill J (2016). Tackling drug-resistant infections globally: final report and recommendations. https://amr-review.org/sites/default/files/160525_Final%20paper_with%20cover.pdf.

[4] O’Neill J (2014). Antimicrobial resistance: tackling a crisis for the health and wealth of nations the review on antimicrobial resistance. https://amr-review.org/sites/default/files/AMR%20Review%20Paper%20-%20Tackling%20a%20crisis%20for%20the%20health%20and%20wealth%20of%20nations_1.pdf.

[5] (2019). Antibiotic resistance threats in the United States, 2019. https://stacks.cdc.gov/view/cdc/82532.

[6] Naylor NR, Atun R, Zhu N (2018). Estimating the burden of antimicrobial resistance: a systematic literature review. Antimicrob Resist Infect Control.

[7] Cassini A, Högberg LD, Plachouras D (2019). Attributable deaths and disability-adjusted life-years caused by infections with antibiotic-resistant bacteria in the EU and the European Economic Area in 2015: a population-level modelling analysis. Lancet Infect Dis.

[8] Migliara G, Di Paolo C, Barbato D (2019). Multimodal surveillance of healthcare associated infections in an intensive care unit of a large teaching hospital. Ann Ig.

[9] Murray CJL, Ikuta KS, Sharara F (2022). Global burden of bacterial antimicrobial resistance in 2019: a systematic analysis. The Lancet.

[10] (2024). WHO Bacterial Priority Pathogens List, 2024: bacterial pathogens of public health importance to guide research, development and strategies to prevent and control antimicrobial resistance.

[11] Hay SI, Rao PC, Dolecek C (2018). Measuring and mapping the global burden of antimicrobial resistance. BMC Med.

[12] Tacconelli E, Sifakis F, Harbarth S (2018). Surveillance for control of antimicrobial resistance. Lancet Infect Dis.

[13] European Centre for Disease Prevention and Control (2023). Antimicrobial resistance in the EU/EEA (EARS-net) - annual epidemiological report 2022. Stockholm.

[14] (2018). Emerging antimicrobial resistance reporting: guide for emerging AMR event sharing. https://www.who.int/publications/i/item/9789241514583.

[15] (2021). EpiPulse - the European surveillance portal for infectious diseases. European Centre for Disease Prevention and Control.

[16] (2022). Global antimicrobial resistance and use surveillance system (GLASS) report 2022.

[17] (2023). GLASS manual for antimicrobial resistance surveillance in common bacteria causing human infection. World Health Organization.

[18] Global action plan on antimicrobial resistance. World Health Organization.

[19] (2018). GLASS emerging antimicrobial resistance reporting framework (GLASS-EAR). World Health Organization.

[20] Sulis G, Sayood S, Gandra S (2022). Antimicrobial resistance in low- and middle-income countries: current status and future directions. Expert Rev Anti Infect Ther.

[21] Gandra S, Alvarez-Uria G, Turner P, Joshi J, Limmathurotsakul D, van Doorn HR (2020). Antimicrobial resistance surveillance in low- and middle-income countries: progress and challenges in eight South Asian and Southeast Asian countries. Clin Microbiol Rev.

[22] (2023). Implementing the global action plan on antimicrobial resistance: first quadripartite biennial report. https://www.who.int/publications/i/item/9789240074668.

[23] Iera J, Seghieri C, Tavoschi L (2023). Early warning systems for emerging profiles of antimicrobial resistance in Italy: a national survey. Int J Environ Res Public Health.

[24] Vong S, Anciaux A, Hulth A (2017). Using information technology to improve surveillance of antimicrobial resistance in South East Asia. BMJ.

[25] About CDC’s AR lab network. Centers for Disease Control and Prevention.

[26] Higgins JPT, Thomas J, Chandler J, Cumpston M, Li T, Page MJ (2021). Cochrane handbook for systematic reviews of interventions version 6.2 (updated February 2021).

[27] Page MJ, McKenzie JE, Bossuyt PM (2021). The PRISMA 2020 statement: an updated guideline for reporting systematic reviews. BMJ.

[28] Fantom NJ, Serajuddin U The World Bank’s classification of countries by income. Policy research working paper, no. WPS 7528 Washington, D.C. World Bank Group.

[29] Turnidge JD, Meleady KT (2018). Antimicrobial Use and Resistance in Australia (AURA) surveillance system: coordinating national data on antimicrobial use and resistance for Australia. Aust Health Rev.

[30] Tsutsui A, Suzuki S (2018). Japan nosocomial infections surveillance (JANIS): A model of sustainable national antimicrobial resistance surveillance based on hospital diagnostic microbiology laboratories. BMC Health Serv Res.

[31] Suzuki S (2021). A view on 20 years of antimicrobial resistance in Japan by two national surveillance systems: the national epidemiological surveillance of infectious diseases and Japan nosocomial infections surveillance. Antibiotics (Basel).

[32] Johnson AP (2015). Surveillance of antibiotic resistance. Philos Trans R Soc Lond B Biol Sci.

[33] Kajihara T, Yahara K, Hirabayashi A, Shibayama K, Sugai M (2021). Japan Nosocomial Infections Surveillance (JANIS): current status, international collaboration, and future directions for a comprehensive antimicrobial resistance surveillance system. Jpn J Infect Dis.

[34] National alert system for critical antimicrobial resistances (caralert). Australian Commission on Safety and Quality in Health Care.

[35] One-health surveillance of antimicrobial resistance in Canada: spotlight on AMRNet. Government of Canada.

[36] E-sin: signalement externe des infections nosocomiales [Article in French]. Santé publique France.

[37] Un système d’alerte: le signalement externe des infections associées aux soins [Article in French]. Santé publique France.

[38] Les bhre, bactéries hautement Résistantes émergentes [Article in French]. Centre d’appui pour la prévention des infections associées aux soins.

[39] Multisector surveillance of antibiotic sales and resistance. The Public Health Agency of Sweden.

[40] UK public health antimicrobial resistance alerts. Public Health England.

[41] Antibiotic resistance laboratory network. Centers for Disease Control and Prevention.

[42] How labs work together. Centers for Disease Control and Prevention.

[43] Rudnick W, Mukhi SN, Reid-Smith RJ (2022). Overview of Canada’s Antimicrobial Resistance Network (AMRNet): A data-driven One Health approach to antimicrobial resistance surveillance. Can Commun Dis Rep.

[44] (2022). National alert system for critical antimicrobial resistances (CARAlert) laboratory handbook. Australian Commission on Safety and Quality in Health Care.

[45] (2022). CARAlert standard operating procedures. https://www.safetyandquality.gov.au/publications-and-resources/resource-library/caralert-standard-operating-procedures.

[46] Doshi N (2022). Bacteriology reference department user manual. GOV-13422.

[47] Canadian antimicrobial resistance surveillance system (CARSS) report 2022. Government of Canada.

[48] Berger-Carbonne A (2024). La lettre du signalement. Infections associées aux soins.

[49] Actualisation des recommandations relatives à la maîtrise de la diffusion des bactéries hautement résistantes aux antibiotiques émergentes (BHRe) [Article in French]. Conseil de la santé publique.

[50] (2022). National action plan for combating antibiotic-resistant bacteria. Year 5 report.

[51] (2014). Swedish work on containment of antibiotic resistance. Tools, methods and experiences.

[52] Aspevall O, Dobric S, Jagdmann J, Nilsson O, Pringle M A report on Swedish antibiotic sales and resistance in human medicine (Swedres) and Swedish veterinary antibiotic resistance monitoring (Svarm) editors. Swedish Veterinary Agency.

[53] AURA 2023: fifth Australian report on antimicrobial use and resistance in human health. Australian Commission on Safety and Quality in Health Care.

[54] CARAlert annual report: 2023. Australian Commission on Safety and Quality in Health Care.

[55] (2001). Updated guidelines for evaluating public health surveillance systems recommendations from the guidelines working group. Centers for Disease Control and Prevention.

[56] (2021). Emergence and increase of new combinations of carbapenemases in enterobacterales in Latin America and the Caribbean. Pan American Health Organization.

[57] Lessa FC, Sievert DM (2023). Antibiotic resistance: a global problem and the need to do more. Clin Infect Dis.

[58] Saltman RB, Bankauskaite V, Vrangbaek K (2007). Decentralization in Health Care: Strategies and Outcomes.

[59] James C, Beazley I, Penn C, Philips L, Dougherty S (2019). Decentralisation in the health sector and responsibilities across levels of government. OECD J Budget.

[60] Bonomo RA, Burd EM, Conly J (2018). Carbapenemase-producing organisms: a global scourge. Clin Infect Dis.

[61] (2023). Embracing a one health framework to fight antimicrobial resistance. Organisation for Economic Co-operation and Development (OECD).

[62] ECDC public health microbiology strategy 2018 – 2022. European Centre for Disease Prevention and Control (ECDC).

[63] (2022). Piano nazionale di contrasto all’Antimicrobico-resistenza (PNCAR) 2022-2025 [Article in Italian]. https://www.salute.gov.it/imgs/C_17_pubblicazioni_3294_allegato.pdf.

[64] New Zealand antimicrobial resistance action plan. Ministry of Health.

[65] (2017). Ireland’s national action plan on antimicrobial resistance 2017 - 2020. https://assets.gov.ie/9519/afcba9bce7c54bf9bcbe9a74f49fdaf2.pdf.

[66] Digital technologies for infectious disease surveillance, prevention and control - a scoping review of the research literature 2015−2019. https://www.ecdc.europa.eu/en/publications-data/digital-technologies-surveillance-prevention-and-control-infectious-diseases.

[67] Brownstein JS, Rader B, Astley CM, Tian H (2023). Advances in artificial intelligence for infectious-disease surveillance. N Engl J Med.

[68] Sakagianni A, Koufopoulou C, Feretzakis G (2023). Using machine learning to predict antimicrobial resistance-a literature review. Antibiotics (Basel).

[69] MacIntyre CR, Chen X, Kunasekaran M (2023). Artificial intelligence in public health: the potential of epidemic early warning systems. J Int Med Res.

[70] (2022). A One Health Priority Research Agenda for Antimicrobial Resistance.

[71] Adisasmito WB, Almuhairi S, One Health High-Level Expert Panel (OHHLEP) (2022). One Health: a new definition for a sustainable and healthy future. PLoS Pathog.

[72] Loftus M, Stewardson A, Naidu R (2020). Antimicrobial resistance in the Pacific Island countries and territories. BMJ Glob Health.

[73] Núñez-Núñez M, Navarro MD, Palomo V (2018). The methodology of surveillance for antimicrobial resistance and healthcare-associated infections in Europe (SUSPIRE): a systematic review of publicly available information. Clin Microbiol Infect.

