# Supplementary material for: Availability and Key Characteristics of National Early Warning Systems for Emerging Profiles of Antimicrobial Resistance in High-Income Countries: Systematic Review
Source: JMIR Public Health Surveill. 2025 Jan 15;11:e57457. doi: 10.2196/57457 (PMC11753579; doi:10.2196/57457)
Supplement: Multimedia Appendix 1 [file publichealth-v11-e57457-s001.pdf]

Supplementary Table S1. Search strategies used in the systematic review.

Pubmed	(“emerging antibiotic resistance” OR “emerging antimicrobial resistance” OR “unusual antibiotic resistance” OR “unusual antimicrobial resistance” OR “atypical antibiotic resistance” OR “atypical antimicrobial resistance” OR “emerging antibiotic resistant” OR “emerging antimicrobial resistant” OR “unusual antibiotic resistant” OR “unusual antimicrobial resistant” OR “atypical antibiotic resistant” OR “atypical antimicrobial resistant” OR “pan-drug resistance” OR “pan-drug resistant” OR “PDR” OR “extensively drug-resistance” OR “extensively drug-resistant” OR “XDR” OR “extreme drug-resistant” OR “novel antibiotic resistance” OR “new antibiotic resistance” OR “novel antibiotic resistant” OR “new antibiotic resistant” OR “critical resistan*” OR "Drug Resistance, Microbial"[Mesh]) AND (“surveillance system” OR “biosurveillance system” OR “alert system” OR “early warning system” OR “laboratory-based surveillance” OR “syndromic surveillance” OR “sentinel surveillance” OR “integrated surveillance” OR “sentinel health event” OR “sentinel health events” OR “surveillance systems” OR “biosurveillance systems” OR “alert systems” OR “early warning systems” OR "Public Health Surveillance"[Mesh] OR "Sentinel Surveillance"[Mesh])
Scopus	TITLE-ABS-KEY(“emerging antibiotic resistance” OR “emerging antimicrobial resistance” OR “unusual antibiotic resistance” OR “unusual antimicrobial resistance” OR “atypical antibiotic resistance” OR “atypical antimicrobial resistance” OR “emerging antibiotic resistant” OR “emerging antimicrobial resistant” OR “unusual antibiotic resistant” OR “unusual antimicrobial resistant” OR “atypical antibiotic resistant” OR “atypical antimicrobial resistant” OR “pan-drug resistance” OR “pan-drug resistant” OR “PDR” OR “extensively drug-resistance” OR “extensively drug-resistant” OR “XDR” OR “extreme drug-resistant” OR “novel antibiotic resistance” OR “new antibiotic resistance” OR “novel antibiotic resistant” OR “new antibiotic resistant” OR “critical resistan*” OR “antimicrobial resistan*” OR “antibiotic resistan*”) AND TITLE-ABS-KEY(“surveillance system” OR “biosurveillance system” OR “alert system” OR “early warning system” OR “laboratory-based surveillance” OR “syndromic surveillance” OR “sentinel surveillance” OR “integrated surveillance” OR “sentinel health event” OR “sentinel health events” OR “surveillance systems” OR “biosurveillance systems” OR “alert systems” OR “early warning systems”)
Web of Science	TS=(“emerging antibiotic resistance” OR “emerging antimicrobial resistance” OR “unusual antibiotic resistance” OR “unusual antimicrobial resistance” OR “atypical antibiotic resistance” OR “atypical antimicrobial resistance” OR “emerging antibiotic resistant” OR “emerging antimicrobial resistant” OR “unusual antibiotic resistant” OR “unusual antimicrobial resistant” OR “atypical antibiotic resistant” OR “atypical antimicrobial resistant” OR “pan-drug resistance” OR “pan-drug resistant” OR “PDR” OR “extensively drug-resistance” OR “extensively drug-resistant” OR “XDR” OR “extreme drug-resistant” OR “novel antibiotic resistance” OR “new antibiotic resistance” OR “novel antibiotic resistant” OR “new antibiotic resistant” OR “critical resistan*” OR “antimicrobial resistan*” OR “antibiotic resistan*”) AND TS=(“surveillance system” OR “biosurveillance system” OR “alert system” OR “early warning system” OR “laboratory-based surveillance” OR “syndromic surveillance” OR “sentinel surveillance” OR “integrated surveillance” OR “sentinel health event” OR “sentinel health events” OR “surveillance systems” OR “biosurveillance systems” OR “alert systems” OR “early warning systems”)

Supplementary Table S2. High-income countries institutional websites explored.

Country	Institution	Websites
American Samoa	Ministry of Health	https://www.health.gov.ws/
Andorra	Ministry of Health	https://www.salut.ad/
Antigua and Barbuda	Ministry of Health, Wellness and the Environment	https://health.gov.ag/
Aruba	National Institute for Public Health and Environment<br><br>Ministry of Health Welfare and Sport (Netherlands)	https://www.rivm.nl/isis-ar/overzichten-van-gegevens/nethmap
Australia	Australian Commission on Safety and Quality in Health Care	https://www.safetyandquality.gov.au/
Austria	Ministry of Health	https://www.sozialministerium.at/en.html
Bahamas, The	Ministry of Health and Wellness	http://www.bahamas.gov.bs/health
Bahrain	Ministry of Health	https://www.moh.gov.bh/?lang=en
Barbados	Ministry of Health and Wellness	https://www.health.gov.bb/#
Belgium	Federal Public Service Health, Food Chain Safety and Environment	https://www.health.belgium.be/en
Bermuda	Ministry of Health	https://www.gov.bm/ministry/health
British Virgin Islands	Ministry of Health and Social development	http://www.bvi.gov.vg/content/ministry-health-and-social-development
Brunei Darussalam	Ministry of Health	https://www.moh.gov.bn/
Canada	Public Health Agency of Canada<br><br>Government	https://www.canada.ca/en/public-health.html<br><br>https://www.canada.ca/en.html
Cayman Islands	Ministry of Health and Wellness	https://www.gov.ky/health-wellness/
Channel Islands	NA	NA
Chile	Ministry of Health	https://www.gob.cl/en/ministries/ministry-of-health/<br><br>https://www.minsal.cl/
Croatia	Ministry of Health<br><br>Institute of Public Health	https://zdravlje.gov.hr/<br><br>https://www.hzjz.hr/en/about-us/
Curaçao	NA	NA

Cyprus	Ministry of Health	https://www.moh.gov.cy/moh/moh.nsf/index_en/index_en?OpenDocument
Czechia*	Ministry of Health	https://www.mzcr.cz/en/the-ministry-of-health/
Denmark	Ministry of Health	https://www.danmap.org/
Estonia	Ministry of Social Affairs	https://www.sm.ee/en
Faroe Islands	Ministry of Health	https://www.hmr.fo/en/what-we-do/health-and-prevention
Finland	Ministry of Social Affairs and Health	https://stm.fi/en/frontpage
France	Ministry of Health	https://solidarites-sante.gouv.fr/
French Polynesia	NA	NA
Germany	Ministry of Health	https://www.bundesgesundheitsministerium.de/en/topics/antimicrobial-resistance.html
Gibraltar	Government	https://www.gibraltar.gov.gi/health
Greece	Ministry of Health<br>National Public Health Organization	https://www.moh.gov.gr/<br>https://eody.gov.gr/
Greenland	Government	https://naalakkersuisut.gl/
Guam	Government	https://www.guam.gov/
Guyana	Ministry of Health	https://www.health.gov.gy/
Hong Kong Special Administrative Region (SAR), China	Government<br>Centre for Health protection, Department of Health	https://www.chp.gov.hk/en/features/47850.html
Hungary	Ministry of Health	https://2010-2014.kormany.hu/en/ministry-of-human-resources
Iceland	Ministry of Health	https://www.government.is/ministries/ministry-of-health/
Ireland	Ministry of Health<br>Health Protection Surveillance Centre (HPSC)	https://www.gov.ie/en/organisation/department-of-health/<br>https://www.hpsc.ie/abouthpsc/
Isle of Man	Government	https://www.gov.im/antibioticresistance
Israel*	Ministry of Health<br>National Public Health Organization	https://www.gov.il/en/departments/ministry_of_health/govil-landing-page<br>http://www.gertnerinst.org.il/e/
Italy	Ministry of Health<br>National Health Institute	https://www.salute.gov.it/portale/antibioticoresistenza/homeAntibioticoResistenza.jsp<br>https://www.epicentro.iss.it/antibiotico-resistenza/ar-iss
Japan	Ministry of Health, Labour and Welfare	https://www.mhlw.go.jp/english/<br>https://janis.mhlw.go.jp/english/about/

Korea, Rep.	Ministry of Health<br><br>National Public Health Organization	http://www.mohw.go.kr/eng/<br><br>https://www.kdca.go.kr/index.es?sid=a3
Kuwait	National Public Health Organization	http://www.hsc.edu.kw/
Latvia	Ministry of Health<br><br>National Public Health Organization	https://www.vm.gov.lv/lv<br><br>https://bior.lv/en
Liechtenstein	Government	https://www.regierung.li/default.aspx?lang=en
Lithuania	Ministry of Health	https://sam.lrv.lt/en/
Luxembourg	Ministry of Health<br><br>National Public Health Organization<br><br>National Laboratory of Health	https://msan.gouvernement.lu/fr.html<br><br>https://www.lih.lu/en/<br><br>https://lns.lu/en/?doing_wp_cron=1688649309.6410770416259765625000
Macao SAR, China	Government	https://www.gov.mo/en/entity-page/entity-552/
Malta	Ministry of Health	https://health.gov.mt/#
Monaco	Government	https://www.gouv.mc/Gouvernement-et-Institutions/Le-Gouvernement/Departement-des-Affaires-Sociales-et-de-la-Sante
Nauru	NA	NA
Netherlands	National Institute for Public Health and the Environment<br><br>Ministry of Health Welfare and Sport	https://www.rivm.nl/en<br><br>https://www.rivm.nl/surveillance-van-infectieziekten/infectieziekten-surveillance-informatie-systeem-antibiotica-resistentie-isis-ar
New Caledonia	Government	https://dass.gouv.nc/
New Zealand	Institute of Environmental Science and Research (ESR)<br><br>Ministry of Health	https://www.esr.cri.nz/<br>https://www.health.govt.nz/
Northern Mariana Islands	NA	NA
Norway	Norwegian Institute of Public Health	https://www.fhi.no/en/in/surveillance/norm/norwegian-surveillance-system-for-antimicrobial-drug-resistance-norm/<br><br>https://www.fhi.no/contentassets/c183b18ccc4a4005a6b9cfae28c97351/norm-norm-vet-2021.pdf
Oman	NA	NA
Panama	Ministry of Health	https://www.minsa.gob.pa/
Poland	NA	NA
Portugal	Ministry of Health	https://www.dgs.pt/

Puerto Rico	Centers for Disease Control and Prevention	https://www.cdc.gov/drugresistance/laboratories.html
Qatar	Ministry of Public Health	https://www.moph.gov.qa/english/Pages/default.aspx
Romania	Ministry of Health	https://www.ms.ro/ro/
San Marino	NA	NA
Saudi Arabia*	Ministry of Health	https://www.moh.gov.sa/Pages/Default.aspx
Seychelles	Ministry of Health	http://www.health.gov.sc/
Singapore	National Centre for Infectious Diseases' National Public Health	https://www.ncid.sg/Health-Professionals/Pages/Antimicrobial-Resistance.aspx<br><br>https://www.ncid.sg/Health-Professionals/Documents/One%20Health%20Report%20on%20Antimicrobial%20Utilisation%20and%20Resistance%202019.pdf
Sint Maarten (Dutch part)	Ministry of Health	https://www.sintmaartengov.org/Ministries/Departments/Pages/Department-of-Public-Health.aspx
Slovak Republic	NA	NA
Slovenia	Ministry of Health	https://www.gov.si/en/state-authorities/ministries/ministry-of-health/
Spain	Ministry of Health	https://www.sanidad.gob.es/en/home.htm
St. Kitts and Nevis	Government	https://www.gov.kn/
St. Martin (French part)	Government	http://www.sintmaartengov.org/government/VSA/Pages/default.aspx
Sweden	Public Health Agency of Sweden	https://www.folkhalsomyndigheten.se/the-public-health-agency-of-sweden/communicable-disease-control/antibiotics-and-antimicrobial-resistance/surveillance-of-antibacterial-resistance/
Switzerland	Federal Office of Public Helath	https://www.bag.admin.ch/bag/en/home.html
Taiwan, China	Taiwan Centers for Disease Control	https://www.cdc.gov.tw/En
Trinidad and Tobago	Ministry of Health	https://health.gov.tt/
Turks and Caicos Islands	Ministry of Health	https://www.gov.tc/moh/
United Arab Emirates	Ministry of Health and Prevention	https://mohap.gov.ae/en
United Kingdom	Government	https://www.gov.uk/health-and-social-care/health-protection
United States	Centers for Disease Control and Prevention	https://www.cdc.gov/drugresistance/laboratories.html

Uruguay	Ministry of Health	https://www.gub.uy/ministerio-salud-publica/
Virgin Islands (U.S.)	Ministry of Health	https://doh.vi.gov/

NA, not available; * language restriction
